# Role of Phytonutrients in Nutrigenetics and Nutrigenomics Perspective in Curing Breast Cancer

**DOI:** 10.3390/biom11081176

**Published:** 2021-08-09

**Authors:** Tanima Bhattacharya, Soumam Dutta, Rokeya Akter, Md. Habibur Rahman, Chenmala Karthika, Hechanur Puttappa Nagaswarupa, Hanabe Chowdappa Ananda Murthy, Ovidiu Fratila, Roxana Brata, Simona Bungau

**Affiliations:** 1 College of Chemistry and Chemical Engineering, Hubei University, Wuhan 430062, China; btanima1987@gmail.com; 2 Techno India NJR Institute of Technology, Udaipur, Rajasthan 313003, India; 3 Food and Nutrition Division, University of Calcutta, Calcutta 700027, India; soumam_dutta@yahoo.com; 4 Department of Pharmacy, Jagannath University, Sadarghat, Dhaka 1100, Bangladesh; rokeyahabib94@gmail.com; 5 Department of Global Medical Science, Yonsei University Wonju College of Medicine, Yonsei University, Wonju 26426, Gangwon-do, Korea; 6 Department of Pharmacy, Southeast University, Banani, Dhaka 1213, Bangladesh; 7 Department of Pharmaceutics, JSS College of Pharmacy, JSS Academy of Higher Education & Research, Ooty 643001, India; karthika1994haridas@gmail.com; 8 Department of Studies in Chemistry, Davangere University, Shivagangothri, Davangere 577007, India; nswarupa@davangereuniversity.ac.in; 9 Department of Applied Chemistry, School of Applied Natural Science, Adama Science and Technology University, Adama P.O. Box 1888, Ethiopia; anandkps350@gmail.com; 10 Department of Medical Disciplines, Faculty of Medicine and Pharmacy, University of Oradea, 410073 Oradea, Romania; ovidiufr@yahoo.co.uk (O.F.); roxana.gavrila@yahoo.com (R.B.); 11 Department of Pharmacy, Faculty of Medicine and Pharmacy, University of Oradea, 410028 Oradea, Romania; 12 Doctoral School of Biological and Biomedical Sciences, University of Oradea, 410087 Oradea, Romania

**Keywords:** breast cancer, phytonutrients, chemosensitizer, polyphenols, nutrigenomic, gene expression, natural compounds

## Abstract

Breast cancer (BC) is one of the most common type of cancer and an important contributor to female mortality. Several genes and epigenetic modifications are involved in the development and progression of BC. Research in phytochemistry, nutrigenomics, and nutrigenetics has provided strong evidence that certain phytonutrients are able to modulate gene expression at transcriptional and post-transcriptional levels. Such phytonutrients may also be beneficial to prevent and treat BC. In this review, we will focus on the nutrigenomic effects of various phytochemicals including polyphenols, phytosterols, terpenoids, alkaloids, and other compounds from different sources. Overall, these phytonutrients are found to inhibit BC cell proliferation, differentiation, invasion, metastasis, angiogenesis, and induce apoptotic cell death by targeting various molecular pathways. They also alter epigenetic mechanisms and enhance the chemosensitivity and radiosensitivity of cancer cells. Such phytochemicals may be used for the effective management of BC patients in the clinical setting in the future. The present article aims to summarize the specific molecular pathways involved in the genetic effects of phytochemicals in BC.

## 1. Introduction

Breast cancer (BC) is one of the most common causes of female mortality around the globe. It is the second most common cancer and the fifth leading cause of death from cancer in the world [1]. It accounts for around 25% of all female cancers [2]. The worldwide incidence of BC in 2012 was 1.67 million, which is alarming [3]. The incidence may increase to 3.2 million by 2050 [4,5]. Males may also develop BC, but this is very rare, accounting for <1% of diagnosed BCs worldwide [6]. BC may be of different types, based on various factors including etiology, location, and clinical and molecular characteristics. Based on location, BC may be of two types; namely, non-invasive and invasive [7]. Non-invasive BC does not extend away from the lobules or duct where it is located. Invasive BC, on the other hand, reaches out from the lobules and ducts to the nearby mammary tissue [7].

BC may be further classified based on the expression of estrogen receptors (ER) into two broad groups; namely, ER-positive and ER-negative [8]. Other molecular characteristics, such as expression of progesterone receptors (PR) [9] and human epidermal growth factor receptor 2 (HER2), [10] are also used for categorization of BC. Such hormone receptor positive BCs maybe treated with aromatase inhibitors or hormonal therapy [11]. BCs that do not exhibit increased expression of any of these three hormone receptors are known as triple negative BCs (TNBCs) [12]. TNBC does not respond to hormonal therapy. 

The common approaches used in the management of BCs include chemotherapy, radiotherapy, and surgical interventions, which often results in significant side effects [13]. Increase in drug resistance further limits the therapeutic potentials of many of these interventions. Thus, researchers are continuously searching for novel alternative strategies for dealing with such conditions in a more effective way. 

Long before the discovery of modern medicine, herbal remedies were used by ancient people for managing a wide range of diseases under traditional medicine systems, such as Chinese medicine, Tibetan medicine, Ayurveda, Siddha, Unani, Roman medicine, Greek medicine, Mesopotamian medicine, Egyptian medicine [14], etc. Due to their potent biological activities, research on medicinal plants and phytochemistry has gained much importance in recent days. Phytochemicals are unique bioactive organic compounds, which are mostly secondary metabolites obtained from plant sources [15,16]. Almost all plant sources are rich in such phytochemicals, which are involved in defense systems of plants and help in the interaction with the biotic environment. More than 5000 unique phytochemicals have been isolated from various plant sources including fruits, vegetables, grains, traditional herbs, etc. [17]. Much more phytochemicals are yet to be discovered. 

Many of these phytochemicals are being used as pharmaceuticals, nutraceuticals, coloring agents, flavoring agents, food additives, agrochemicals, cosmetics, etc. These compounds exert their biological effects mainly by modulating certain molecular targets including cellular receptors, neuroreceptors, ion channels, ion pumps, cytoskeleton, transcription machinery, etc. [18,19]. Such properties maybe useful for preventing and treating various ailments such as cancer [20,21], diabetes [22,23], heart disease [24], neurodegeneration [25,26], pre- and post-menopausal manifestations [27,28,29], skin disease and wound healing [30], etc. Certain phytochemicals are found to exert anti-BC effects by modulating some genes and signal transduction pathways. These phytochemicals inhibit breast carcinoma mostly by reducing cell proliferation, inducing apoptosis, decreasing metastasis, suppressing angiogenesis, and reducing the migratory properties of cancer cells [31,32]. These compounds are also found to enhance the therapeutic efficiency of other anti-cancer drugs, sensitization to radiation, and prevent drug resistance in cancerous tissue [31]. 

As previously mentioned, phytochemicals are proven natural ingredients with the ability to treat/ameliorate various diseases. In the case of BC, it has been reported that, with the strong activity of phytochemicals, the incidence rate and recurrence of BC could be greatly reduced. This article aims to summarize the specific molecular pathways involved in the genetic effects of phytochemicals in BC. On the other hand, phytochemicals reported for the treatment and management of BC require more supportive clinical data for confirmation. The field of natural substances (of vegetal origin) being very diverse implies the need for extensive studies to confirm the action and therapeutic role of phytochemicals; in consequence, another purpose of this research was also to provide valuable data as the most informative and recent background support necessary for the further development of BC research and treatment.

## 2. Methodology

**Figure 1:**
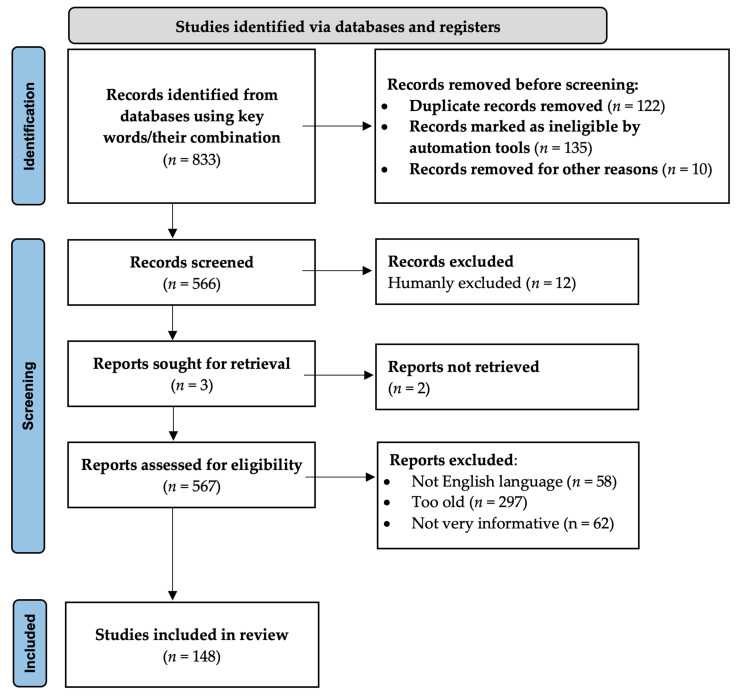
Flow chart presenting the steps of published data selection for being included in the present paper.

In order to select, as carefully as possible, the most relevant articles (available in the most known medical/biology/chemical databases) we refer to in this review, an algorithm imposed by the flow chart presented in Figure 1 (according to Page et al. recommendations [33,34]) was applied, including all the steps/selection criteria for the necessary material in the literature.

## 3. Genetics of Breast Cancer

Several genes are found to be involved in BC, which significantly influence their screening and follow-up strategies. Heredity does play an important role in BC, but <30% of patients with a family history of BC have specific predisposing genes [35]. The majority of hereditary cases (up to 25%) are attributed to the mutations in some specific highly penetrant and rare genes, which confer an 80% life time risk of BC [35]. Such genes include BC genes A1 (*BRCA1*) and A2 (*BRCA2*), phosphatase and tensin homolog (*PTEN*), tumor protein p53 (*TP53*), cadherin-1 (*CDH1*), and serine/threonine kinase-11 (*STK11*) [35]. Specific clinical guidelines are available for the management of such patients. Mutations in some moderately penetrant and rare genes such as checkpoint kinase-2 (*CHEK2*), BRCA1-interacting protein-1 (*BRIP1*), ataxia telangiectasia mutated (*ATM*), and partner and localizer of BRCA2 (*PALB2*) are also involved in the development of BC in a minority of cases (2 to 3%) [35]. Such mutations may increase the risk two-fold. 

**Figure 2:**
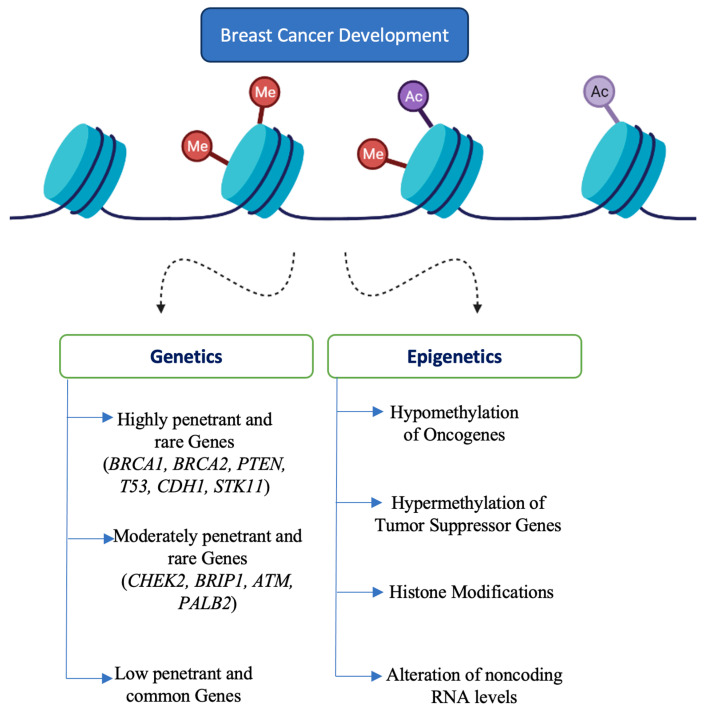
Genetic and epigenetic contributors of breast cancer. Legend: breast cancer gene A (*BRCA*), phosphatase and tensin homolog (*PTEN*), tumor protein p53 (*TP53*), cadherin-1 (*CDH1*) and serine/threonine kinase-11 (*STK11*), checkpoint kinase 2 (*CHEK2*), BRCA1-interacting protein-1 (*BRIP1*), ataxia telangiectasia mutated (*ATM*), partner and localizer of *BRCA2* (*PALB2*).

Additionally, mutations in some low-penetrant and common genes may also be involved [18]. Identification of such minor genes is not performed routinely in the clinical setting. In addition to genetic factors, epigenetic factors may also influence the development of BC. Certain epigenetic factors, including methylation of tumor suppressor genes, hypomethylation of oncogenes and repetitive DNAs, stabilization of repressive chromosome looping, downregulation of tumor suppressing micro RNAs, upregulation of metastamiRs and oncomiRs, altered histone modifications, etc., may lead to tumorigenesis in BC [36,37]. Genetic and epigenetic contributors of BC are represented in Figure 2.

## 4. Nutrigenomic Effects of Phytochemicals in Breast Cancer

Phytochemicals can be classified into different groups based on their chemical compositions, such as polyphenols, phytosterols, terpenoids, alkaloids, and other compounds (including organosulfur compounds, saponins, etc.) [38]. Each of these classes have potent biological activities and can modulate various molecular targets. The present paper will review the genetical effects of these phytochemicals in BC, considering also the correlation with the BC genetic background; however, it is certain that further research needs to be conducted in order to clarify all aspects.

### 4.1. Polyphenols 

Polyphenols are a broad group of organic compounds, which include various bioactive plant metabolites such as phenolic acids, flavonoids, stilbenes, lignans, etc. [39,40]. These compounds possess one or more benzene rings and hydroxyl groups. A wide variation in their structural characteristics is responsible for their health benefits, including anti-carcinogenic, anti-inflammatory, anti-oxidant, anti-proliferative, and anti-angiogenic properties [41]. Polyphenols and certain flavonoids are found to inhibit DNA methyl transferases (DNMT) and histone deacetylases (HDAC), thereby enhancing acetylation and demethylation of tumor suppressor genes that prevent BC proliferation and migration [42]. 

Polyphenols may also inhibit signal transducer and activator of transcription 3 (STAT3), thereby decreasing the transcription of target genes involved in immunosuppression, cell proliferation, cell survival, angiogenesis, and metastasis [26]. Stilbenes are unique polyphenols, which are found to interfere with various molecular mechanisms involved in tumorigenesis. Stilbenes are found to modulate certain signal transduction pathways, which ultimately influences the transcription of genes involved in antioxidant defense, inflammatory response, autophagy, and apoptosis [43,44]. For example, stilbenes may promote the phosphatidylinositol-3 kinase/protein kinase B (PI3K/Akt) pathway, which, in turn, activates nuclear factor erythroid 2 related factor 2 (Nrf2). This Nrf2 binds to the antioxidant response elements (ARE), thereby increasing the transcription of genes involved in antioxidant mechanisms. They can also inhibit IkB kinase (IKK) and, thus, the nuclear factor kappa B (NF-kB) pathway involved in the transcription of pro-inflammatory factors, cell proliferation, and survival related genes [45]. Stilbenes may also promote cellular apoptosis by activating caspase 3/7 [45].

### 4.2. Phytosterol

Phytosterols are steroidal alcohols containing 28 or 29 carbon atoms, which are key components of plant plasma membrane [46]. These are not synthesized in the human body and are mostly derived from dietary sources, especially plants that are rich in lipids. Chemically, they act as antioxidants and, physically, as membrane stabilizers [47]. A wide variety of phytosterols are present in the diet, but the most abundant ones are β-sitosterol, camp sterols, ergosterols, and stigma sterols [48,49]. AMP-activated protein kinase (AMPK) is a unique target for treating many forms of cancer, including BC; it acts as a sensor of cellular energy stress, thereby promoting cellular catabolic pathways and inhibiting cellular anabolic pathways, growth, and proliferation. The effects are exerted at both transcriptional and post-transcriptional levels [28]. 

Certain phytosterols (including β-sitosterol) can act as AMPK activators, thereby helping in tumor suppression and cancer prevention. Phytosterols also inhibit the translocation of NF-kB to the nucleus, thereby preventing the expression of pro-inflammatory genes [46]. Phytosterols can also activate liver X receptors (LXRs), which, in turn, may suppress transcription and translation of estrogen receptor alpha, cyclin A2, cyclin D1, and Skp2 proteins, and may enhance p53 expression, which ultimately contribute to the anti-proliferative effects. LXR activation also activates some key lipogenic genes [47,48]. Such unique properties of phytosterols make them potential therapeutic agents against BC [50].

### 4.3. Terpenoids 

Terpenoids are a wide group of bioactive organic compounds derived from plant sources. Based on their number of cyclic structures, they can be broadly classified as hemiterpenoids, monoterpenoids, sesquiterpenoids, diterpenoids, triterpenoids, tetraterpenoids, and polyterpenoids [51]. There unique structures are responsible for a wide range of biological activities such as antioxidant, anti-inflammatory, and anti-carcinogenic effects [52]. The terpenoids are able to prevent cancer cell proliferation and induce apoptosis in cancerous cells via various complex molecular mechanisms. Higher levels of proteasomal activities can be observed in tumor cells, which degrade various proteins involved in cell cycle regulation and apoptosis [51]. 

Terpenoids exert anti-proteasomal activities, thereby preventing abnormal proteasomal functions. Terpenoids are also found to inhibit the NF-kB pathway, which, in turn, reduces the expression of genes involved in pro-inflammatory compound production, cell proliferation, tumor cell invasion, cellular survival, metastasis, and angiogenesis [51,52]. Additionally, terpenoids may downregulate anti-apoptotic B-cell lymphoma-2 (Bcl-2) protein and upregulate pro-apoptotic Bcl-2-associated X (Bax) protein, which ultimately leads to the release of cytochrome c and caspase activation, resulting in cellular apoptosis [51]. Single or a combination of terpenoids maybe used efficiently for managing BC.

### 4.4. Alkaloids 

**Figure 3:**
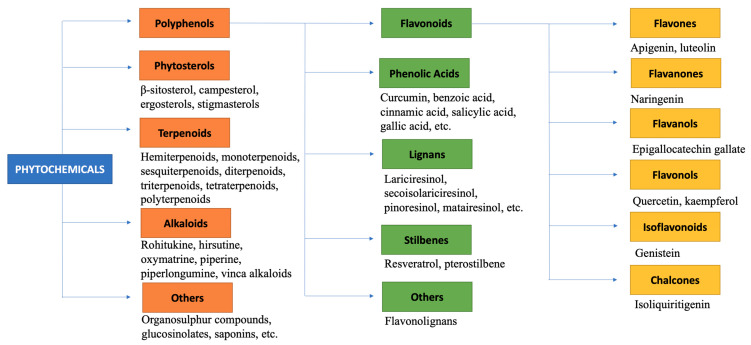
Classification of phytochemicals.

**Figure 4:**
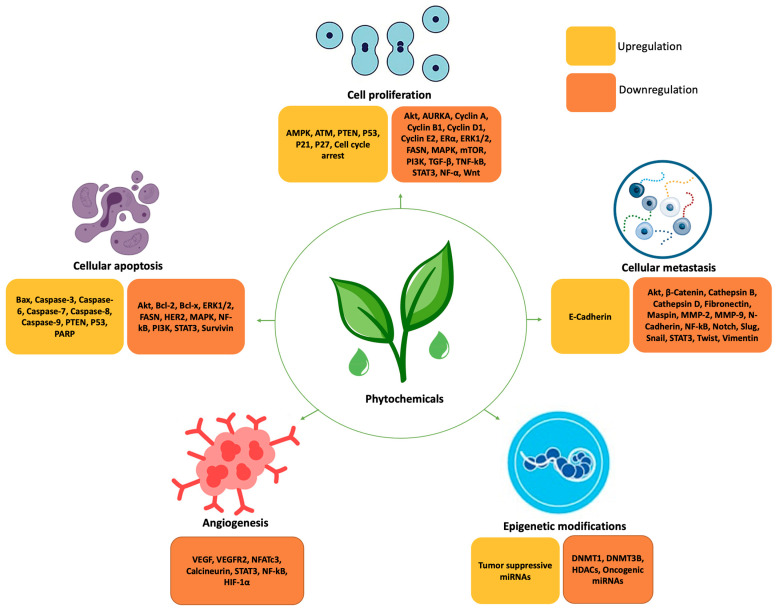
Influence of phytochemicals on gene expression in breast cancer. Phosphatase and tensin homolog (PTEN), poly (ADP-ribose) polymerase (PARP), tumor protein p53 (*TP53*), cadherin-1 (*CDH1*) and serine/threonine kinase-11 (*STK11*), checkpoint kinase 2 (CHEK2), ataxia telangiectasia mutated (*ATM*), sterol regulatory element-binding protein 1 (SREBP-1), B-cell lymphoma-2 (Bcl-2), Bcl-2-associated X (Bax, phosphatidylinositol-3 kinase/protein kinase B (PI3K/Akt), nuclear factor erythroid 2 related factor 2 (Nrf2), signal transducer and activator of transcription 3 (STAT3), AMP-activated protein kinase (AMPK), Aurora protein kinase (AURKA), polo-like kinase-1 (PLK1),human epidermal growth factor receptor 2 (HER 2), mitogen-activated protein kinase (MAPK), hypoxia-inducible factor 1α (HIF-1α), fatty acid synthase (FASN), vascular endothelial growth factor (VEGF), VEGF receptor 2 (VEGFR2), nuclear factor of activated T cells 3 (NFATc3), nuclear factor kappa B (NF-kB), DNA cytosine-5-methyltransferase 1 (DNMT1), histone deacetylases (HDACs), microRNAs (miRNAs), extracellular signal-regulated kinase 1/2 (ERK1/2), transforming growth factor β (TGF- β), transforming growth factor kB (TGF-kB).

Alkaloids are biologically active organic compounds, which may act as potential anti-cancer agents. They are found to increase cytotoxicity, induce DNA damage, modulate survival pathways, increase caspase activity, promote apoptotic cell death, cause cell cycle arrest, and may suppress the NF-kB pathway [38,53]. Certain alkaloids may exert protective effects in BC by targeting several molecular pathways. The alkaloid Rohitukine may increase reactive oxygen species (ROS) levels in BC cells, leading to DNA damage [34]. Hirsutine may also lead to DNA damage, the downregulation of the Akt pathway, and target HER2 proteins [54,55]. Oxymatrine may upregulate Bax and downregulate Bcl-2 and Wnt/β-catenin signaling in BC cells [56,57]. Piperine may induce G1/S and G2/M cell cycle arrest and apoptosis, and inhibit Akt, NF-kB, sterol regulatory element-binding protein 1 (SREBP-1), fatty acid synthase mRNA, HER2, and Matrix metalloproteinase-2 and -9 (MMP-2 and -9) mRNA levels [58]. Piperlongumine may inhibit STAT3, survivin, Bcl-2, and Bcl-x, and upregulate p53 [59]. The vinca alkaloids (including vincristine and vinblastine) are found to exhibit anti-mitotic and anti-microtubule properties [38]. They may induce apoptosis, upregulate caspase expression, and downregulate cyclin D1, leading to cell death [38]. Classification of phytochemicals and their influence on gene expression on BC is given in Figure 3 and Figure 4, respectively.

## 5. Nutrigenomic Effects of Some Selected Phytochemicals in Breast Cancer

We have already noticed that phytochemicals have numerous effects on our genome at various levels, such as transcription, translation, post-translation, etc. Here, we will review the effect of some selected phytochemicals on genetic expression with potent clinical applications in BC. 

### 5.1. Polyphenols

#### 5.1.1. Flavonoids

- Epigallocatechin gallate (EGCG)

EGCG is a unique flavonoid derived mainly from green tea (*Cameillia sinensis*). It is widely studied for its cancer preventing properties and is known to possess anti-oxidant, anti-inflammatory, anti-proliferative, anti-angiogenic, anti-metastatic, anti-genotoxic, apoptotic, and epigenetic effects [60]. It targets various key molecular pathways and modulates the expression of various genes involved in tumorigenesis and tumor progression. EGCG is found to downregulate telomerase, human telomerase reverse transcriptase (hTERT), ERα, and PI3K/Akt and upregulate Bax, p53, caspase 3, caspase 9, and PTEN [61,62,63]. Additionally, it reduces the expression of β-catenin, cyclin D1, and phosphorylated Akt in BC cells [64]. EGCG also has the ability to activate the Nrf-2 pathway and inhibit the NF-kB pathway, Wnt signaling, VEGF, FASN activity, and S-phase kinase-associated protein 2 (Skp2) [38,65]. This flavonoid may alter DNA methylation and cause histone modifications. It reduced the expression of DNMT1, HDAC1, and methyl CpG-binding protein 2 (MeCP2), which are otherwise increased in BC cells [66]. It is also found that EGCG upregulates miR-16 in BC cells, which, when transferred to tumor-associated macrophages (TAMs) through tumor derived exosomes, may inhibit infiltration of TAM and polarization of M2 macrophages necessary for tumor progression [67].

- Genistein

Genistein is a flavonoid with phytoestrogen properties. It is mostly found in soybeans (*Glycine max*). Numerous molecular pathways are targeted by genistein, which leads to its anti-proliferative, anti-inflammatory, anti-metastatic, apoptotic, and cytotoxic effects; it causes cell cycle arrest, reduces cell viability, and improves radiosensitivity [38]. Genistein regulates ERα expression in BC cells. It may repress Erα, thereby reducing cell proliferation and differentiation [68]. It may upregulate Bax and downregulate Bcl-2, leading to apoptosis [38]. Genistein is found to decrease the methylation status and induce the expression of various tumor suppressor genes, including ATM, mammary serpin peptidase inhibitor (SERPINB5), adenomatous polyposis coli (APC), and PTEN in BC cells [69]. It reduces the expression of DNMT1, leading to epigenetic modifications [69]. It also represses cyclin B1, cyclin D1, and induces BRCA1. Additionally, genistein downregulates the NF-kB pathway, the PI3K/Akt pathway, HER2/neu, EGF, VEGF, IGF, platelet derived growth factor (PDGF), fibronectin, angioprotein-2, and cadherin-V and upregulates angiostatin, endostatin, and thrombospondin, leading to its anti-carcinogenic effects [38,70]. Genistein also modulates miRNA levels, such as by the upregulation of miR-23b [71] and downregulation of oncogenic miR-155 [72], resulting in cell death.

- Quercetin

Quercetin is a flavonoid, which is derived from many plant sources and possesses potent anti-cancer properties. Quercetin demonstrates anti-proliferative, anti-metastatic, anti-angiogenic, apoptotic, and chemo-sensitizing effects [38,73]. It is found to repress leptin gene expression, leading to inhibition of T47D cell growth [74]. The compound enhances the expression of Bax and reduces expressions of Bcl-2 proteins in MCF-7 cells. The effect is mostly exerted by involving necroptosis [75]. Quercetin reduces the expression of FASN and β-catenin [76]. It causes cell cycle arrest at Go/G1-phase and may downregulate the expression of survivin, leading to anti-proliferative and apoptotic effects [77]. It represses VEGF, VEGF receptor 2 (VEGFR2), nuclear factor of activated T cells 3 (NFATc3), and the calcineurin pathway, thereby inhibiting angiogenesis [78]. Additionally, it increases the expression of P53 and E-cadherin and reduces expression of mutant P53, vimentin, HER2, and cyclin D1 [38]. It inhibits Twist via the p38 mitogen-activated protein kinase (p38MAPK) pathway, leading to apoptosis in BC cells [79]. It may also inhibit the PI3K/Akt pathway. miR-146a is upregulated by quercetin, which exerts anti-proliferative effects [80]. The ability of quercetin to target such a variety of molecular pathways makes it an ideal lead for anti-cancer drug development.

- Apigenin

Apigenin is a flavonoid (trihydroxyflavone) present in a wide variety of fruits and vegetables including grapefruit, chamomile, parsley, celery, etc. It possesses anti-carcinogenic, anti-inflammatory, and antioxidant properties [38]. It may influence various molecular pathways involved in tumorigenesis. It downregulates the NF-kB pathway involved in inflammation and cell survival. It represses cyclin A, cyclin B, CDK1, p-JAK1, p-JAK2, phosphorylated STAT3, VEGF, MMP-9, TNF-α, Granulocyte macrophage colony stimulating factor (GMCSF), IL-1α, IL-6, p38-MAPK, Akt, and p-HER2 and upregulates, caspase 3, c-PARP, p53, p21, LC3-II [38]. It may also increase the Bcl-2 to Bax ratio [81]. Apigenin may inhibit cell proliferation, invasion, and migration, enhance immune response, and induce apoptosis in BC cells. Apigenin is found to sensitize TNBC spheroids to doxorubicin via targeting heterogeneous ribonuclear protein A2/B1 (hnRNPA2) by enhancing the levels of efflux transporters and apoptosis [82]. Apigenin may increase the cytotoxic effects of doxorubicin by enhancing DNA damage and decreasing the expression of DNA repair genes [83].

- Luteolin

Luteolin is a potent bioactive flavone with anti-cancer properties. It is found in a wide variety of plants, such as celery, parsley, thyme, chamomile tea, etc. Luteolin reduces the expression of VEGF, p-EGFR, p-STAT3, p-Akt, p-ERK1/2, CD44, ALDH, vimentin, slug, β-catenin, cyclin A, cyclin B1, cyclin D1, cyclin E2, MMP-2, MMP-9, Hesfamily BHLH transcription factor 1 (HES1), MAPK, IGF-1, ERα, CDK2, and Bcl-xL and increases the expression of p-21, Bax, p-38, caspase 3, and c-PARP [38]. These effects lead to the suppression of cell proliferation, angiogenesis, metastasis, migration, and cell survival and induce apoptosis in BC cells.

- Kaempferol

Kaempferol is a flavonoid present in many fruits and vegetables. It is a potent antioxidant and exhibits anti-cancer effects due to its anti-proliferative, anti-metastatic, apoptotic, and cytotoxic effects [38]. It may downregulate phosphorylated insulin response substrate-1 (pIRS-1), pAkt, pMEK1/2, pERK1/2, cyclin D1, cyclin E, cathepsin D, cathepsin B, N-cadherin, Snail, Slug, RhoA, Rac1, MMP-9, MMP-2, monocarboxylate transporter 1 (MCT1), glucose transporter 1 (GLUT1) and may upregulate p-53, p21, and E-cadherin, resulting in decreased proliferation, metastasis, migration, and invasion of BC cells [84,85,86]. Additionally, it may induce Bax and poly (ADP-ribose) polymerase (PARP) cleavage and repress Bcl-2, leading to cellular apoptosis [87]. The levels of caspase-3 and caspase-9 are also upregulated [38]. Such unique properties of kaempferol may be utilized for novel drug development.

- Isoliquiritigenin

Isoliquiritigenin is a bioactive chalcone. It can be isolated from the roots of liquorice. It is found to exert anti-cancer effects at various stages of tumorigenesis, including inhibition of cell proliferation, metastasis, angiogenesis, cell cycle arrest, and promotion of apoptosis [88]. It is found to downregulate the NF-kB pathway, PI3K, Akt, Bcl-2, GSK3β, β-catenin, STAT3, DNMT1, Wnt, cyclin D1, survivin, prostaglandin E_2_, VEGF, Hypoxia-inducible factor 1α (HIF-1α), MMP-2, MMP-9, TGF- β, telomerase, and mTOR and may upregulate PTEN, Bax, tumor suppressor gene reversion inducing cysteine rich protein with Kazal motifs 1 (RECK1), Cyt-c, caspase-3, caspase-9, LC3-II, and the proteasomal degradation pathway [38]. The compound also shows chemo-sensitizing properties [38,89]. Isoliquiritigenin is found to alter the expression of certain miRNAs. It may suppress the expression of miR-374a and miR-21, leading to apoptosis, prevention of metastasis, and invasion [38]. As a dietary supplement, isoliquiritigenin was found to induce demethylation of the promoter of WNT inhibitory factor 1 (WIF1) by blocking the catalytic domain of DNMT1, leading to increased WIF1 gene expression and, thereby, halted the cancer development process in mammary tissue via inhibition of BC stem cells [90].

#### 5.1.2. Phenolic Acids

- Curcumin

Curcumin (diferuloylmethane) is a natural polyphenol obtained from the rhizome of turmeric (*Curcuma longa*, Zingiberaceae family). It is responsible for the characteristic yellow color of turmeric. Several studies have confirmed the anti-carcinogenic, anti-proliferator, anti-oxidant, anti-inflammatory, anti-metastatic, anti-angiogenic, apoptotic, radio-protective, and chemo-sensitizing properties of curcumin, which make it an ideal compound for cancer therapy [91,92]. Several gene products (including transcription factors, enzymes, cytokines, and compounds associated with cell proliferation and survival) may be modulated by curcumin, and several cell lines or models for BC have been used for evaluating its effects [38,93,94]. In MCF-7 cell line, curcumin is found to upregulate the expression of caspase-3 and caspase-9. miR-21 is a micro-RNA, which interferes with the translation of many tumor suppressors. Curcumin is found to upregulate phosphatase and tensin homolog/protein kinase B (PTEN/Akt) signaling, which, in turn, downregulates miR-21 expression [43]. Curcumin may also inhibit fatty acid synthase, which may lead to apoptosis in cancer cells [44]. Curcumin reduces methylation of glutathione *S*-transferase Pi 1 (GSTP1) and Ras-association domain family protein 1A (RASSF1A) genes, thereby activating them [93,94]. These are involved in tumor suppression. 

**Figure 5:**
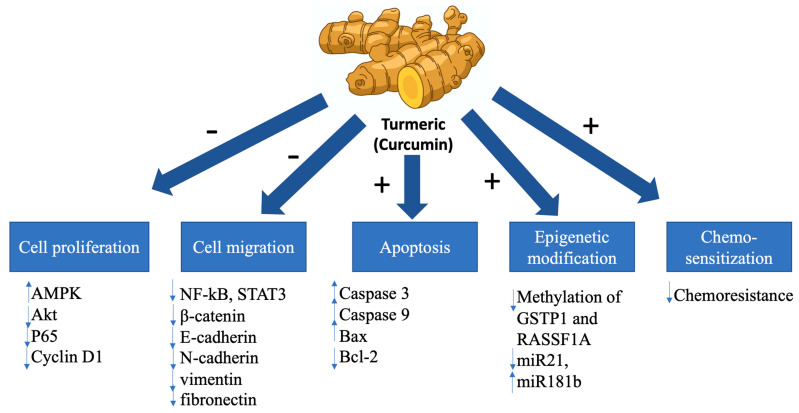
Influence of Curcumin on breast cancer cells. Legend: Ras-association domain family protein 1A (RASSF1A), glutathione *S*-transferase Pi 1 (GSTP1), phosphatase and tensin homolog/protein kinase B, AMP-activated protein kinase (AMPK), signal transducer and activator of transcription 3 (STAT3).

Curcumin also downregulates Bcl-2 and upregulates Bax expression in BC cells, resulting in apoptosis. Additionally, curcumin inhibits NF-kB signaling, STAT3 pathways, and the expression of β-catenin, E-cadherin, N-cadherin, vimentin, fibronectin, and other proteins involved in cell invasion and migration [38]. Figure 5 represents the influence of curcumin on BC.

Additionally, research from previous years has proved that curcumin may have many pharmacological activities (anti-cancer, anti-inflammatory, antioxidant, etc.) without any side effects at dietary intake levels [95]. The targeting of DNA and RNA, but also of intracellular enzymes, is a consequence of the pleiotropic trait manifested consecutively or simultaneously by curcumin molecules [96,97]. 

In the attempt to identify both solubility and bioavailability limits, multiple approaches have been tested. The main strategy approached in the efforts to improve the bioavailability of curcumin has been to modulate the environment in which curcumin is administered. The emergence of new delivery methods (such as liposomes, phospholipid complex, polymeric micelles, microemulsions, nanoparticles, etc.) provides definite possibilities for further exploration in the direction of increasing the curcumin’s oral bioavailability [95]. 

#### 5.1.3. Lignans

- Secoisolariciresinol

Secoisolariciresinol is a lignan with phyto estrogenic properties. It is a biphenolic compound abundantly present in flaxseeds. Phyto estrogenic lignans are turned into enterolignans, such as enterodiol and enterolactone, after consumption. These compounds may bind to and modulate ERs and can be considered selective estrogen receptor modulators (SERMs) [98]. It may downregulate uPA-induced plasmin activation, MMP-2, MMP-9, cyclin A2, cyclin B1, cyclin B2, cyclin E1, phosphorylation of the FAK/paxillin pathway, FASN expression, sex hormone-binding globulin (SHBG), and IGF-binding protein 3 (IGFBP-3) levels and increase cytotoxic effects of chemotherapeutic agents, leading to suppression of cell proliferation, metastasis, migration, cell growth, and tumor progression and promote apoptosis and cell death [38].

#### 5.1.4. Stilbenes

- Resveratrol

Resveratrol is a stilbene and phytoestrogen found in grapes, berries, and peanuts and is known to possess anti-malignant properties [99,100]. It is a plant metabolite produced in response to stressful events and is able to modulate various molecular pathways involved in cell proliferation, apoptosis, metastasis, epigenetic modifications, and chemo-sensitization [101]. Resveratrol is found to upregulate the expression of BRCA1, p53, and p21 and downregulate estrogen receptor α (ERα), cathepsin D, Wnt signaling proteins, and telomerase, leading to inhibition of cell proliferation [38,101]. The compound inhibits DNMTs and HDACs and promotes hypermethylation, leading to epigenetic modification of gene expression [38,101]. It prevents metastasis by downregulating insulin-like growth factor (IGF), epidermal growth factor (EGF), mitogen-activated protein kinase (MAPK), Akt, and PI3K [38,102]. Resveratrol halts the progression of the cell cycle by downregulating Aurora protein kinase (AURKA) and polo-like kinase-1 (PLK1) [50]. It also inhibits other genes involved in cell cycle, angiogenesis, organization of cytoskeleton, and DNA repair in BC cells. The expression of cyclin B1 and cyclin D1 is markedly reduced [103]. It induces the expression of ATPase sarcoplasmic/endoplasmic reticulum Ca^2+^ transporting 3 (ATP2A3) gene, leading to apoptosis and altered intracellular Ca^2+^ concentration in BC cells [104]. In SKBR-3 cells, resveratrol downregulated fatty acid synthase (FASN) and human epidermal growth factor receptor 2 genes (HER-2), resulting in apoptosis [102]. It downregulates phosphorylation of Akt and upregulates PTEN expression, leading to the suppression of the PI3K/Akt/mTOR pathway, which is usually overactive in cancer cells, causing cell proliferation [105]. Additionally, resveratrol may induce Bax, caspase 3, and caspase 9 and repress vascular endothelial growth factor (VEGF) and STAT3, leading to cellular apoptosis [38]. Several tumor suppressive micro RNAs are also regulated by resveratrol, including miR-542-3p, miR-409-3p, miR-200c-3p, miR-125b-5p, and miR-122-5p in BC cells [106].

- Pterostilbene

Pterostilbene is a stilbene found in blueberries. The compound is chemically related to resveratrol. It may downregulate Akt, mTOR, cyclin D1, vimentin, snail, slug, twist 1, zinc finger E-box-binding homeobox 1 (*ZEB1*), glycogen synthase kinase 3β (GSK3β) signaling, the NF-kB pathway, MMP-2, MMP-9, cortactin, membrane type *1-*matrix metalloproteinase (*MT1*-*MMP*), c-Src kinase, and Bcl-2 and may upregulate p-21, Bax, E-cadherin, miR-205 in BC cells, leading to inhibition of cell proliferation, metastasis, and apoptotic cell death [38,107,108,109,110,111]. It may also cause G0/G1 phase arrest in TNBC cells, resulting in suppression of their growth, and enhance apoptosis [107]. 

#### 5.1.5. Flavonolignans

- Silibinin

Silibinin is a unique flavonolignan present as an active compound of silymarin complex extracted from milk thistle seeds. The compound shows anti-cancer properties and is found to exhibit anti-proliferative, anti-metastatic, apoptotic, and chemo-sensitizing properties [38]. It inhibits BC cell metastasis by suppressing chemokine receptor type 4 (CXCR4) [112]. It may also repress the expression of transforming growth factor β2 (TGF- β2), MMP-2, and MMP-9, thereby reducing metastasis in TNBC [113]. Silibinin inhibits phosphorylated extracellular signal-regulated kinases (p-*ERK*) and phosphorylated mitogen-activated protein kinase (p-MEK), thereby inhibiting MMP-9 expression and BC cell migration [114]. Moreover, silibinin represses the expression of ERα gene, hTERT, cyclin D1, Bcl-2, Akt, mTOR, the NF-kB pathway, Wnt signaling, β-catenin, VEGF, EGFR, and cyclooxygenase 2 (COX-2) and induces the expression of Bax, p53, p21, p27, BRCA1, ATM, PTEN, caspase-6, and caspase-9 [38]. All these factors prevent tumor cell proliferation, migration, and viability, reduce tumor volume and infiltration, and promote autophagy, apoptosis, and necrosis. Silibinin also suppress miR-21 and miR-155, leading to tumor suppression [115]. 

### 5.2. Terpenoids

- Thymoquinone

Thymoquinone is a terpene derived from black seed oil with anti-cancer, antioxidant, anti-inflammatory, and cytotoxic properties. It downregulates Wnt, PI3K/Akt, and MAPK and upregulates p53, resulting in apoptosis [116]. It inhibits the expression of VEGF and increases interferon-γ (IFN- γ) levels, thereby suppressing angiogenesis [117]. Additionally, thymoquinone downregulates the NF-kB pathway, Bcl-2, caspase recruitment domain family member 16 (CARD16), EGF-EGFR, G-protein coupled receptor (GPCR), HDAC, p-Akt1, p-65, MMP-2, MMP-9, integrin αV, snail, twist, Smad2, cyclin D1, cyclin E, and survivin and upregulates Bax, protein tyrosine phosphatase receptor-type R (PTPRR), TGF-β, maspin, p21, BRCA1, cytochrome c, procaspase 3, caspase 3, caspase7, caspase 12, PARP, E-cadherin, cytokeratin 19, hypermethylated in cancer 1 (HIC1) gene, and PTEN [38]. The compound is found to induce G2/M phase arrest [118]. It may also alter the methylation status, acetylation status, and miRNA expression, leading to epigenetic modifications [38]. The overall effect of thymoquinone is to inhibit proliferation, metastasis, migration, invasion, viability, and angiogenesis of BC cells, and to promote apoptosis, necrosis, and cell death.

- Parthenolide

Parthenolide is a sesquiterpene lactone, which is found to possess anti-inflammatory and anti-cancer properties [38]. It can be obtained from feverfew herb (*Tanacetum parthenium* L.). In BC cells, parthenolide may induce ROS generation, leading to cell cycle arrest and apoptosis [119]. The compound can suppress the NF-kB pathway causing apoptotic cell death [119]. It may induce autophagy by upregulating Beclin-1 and converting LC3-I to LC3-II. The Akt/mTOR/Nrf2 pathway may be suppressed by parthenolide [38]. Parthenolide may also induce mitochondrial dysfunction and necrosis of cancer stem cells. Moreover, it may enhance the sensitivity of cancer cells to chemotherapy and radiotherapy.

### 5.3. Saponins

- Ginsenosides

Ginsenosides are bioactive saponins present in ginseng roots. These compounds may influence the expression of various target genes involved in BC. Ginsenoside may cause epigenetic modifications of the genes involved in immune response and tumorigenesis. They may increase immune response and inhibit the growth of MCF-7 cells [120]. Hypermethylation of certain genes such as insulin like 5 (INSL5), olfactory receptor family 52 subfamily A member 1 (OR52A1), and caspase 1 (CASP1) leads to their downregulation, whereas hypomethylation of genes such as chromosome 1 open reading frame 198 (C1orf198), ST3 beta-galactoside, alpha-2,3-Sialyltransferase 4 (ST3GAL4), and clathrin interactor 1 (CLINT1) leads to their upregulation [120]. This results in diminished cell proliferation and increased apoptosis. Ginsenosides inhibit the NF-kB pathway and increase caspase 3 and Bax to Bcl-2 ratio, causing apoptosis, chemo-sensitization, and reduced cell proliferation [121]. Ginsenoside Rg1 may induce apoptotic cell death via ROS generation [122]. Additionally, it downregulates MMP-2, MMP-9, mTOR, Akt, JNK, PI3K, VEGFA, VEGFB, VEGFC, miR-18a, Smad2, cyclin D1, cyclin E2, CDK4, survivin, and ERK and upregulates AMPK, LC3-II, p53, caspase 6, caspase 7, caspase 8, caspase 9, p38 MAPKs, and PARP, leading to reduced proliferation, angiogenesis, metastasis, invasion, and enhanced chemo-sensitization and apoptosis [38].

### 5.4. Isothiocyanates

- Benzyl Isothiocyanate

Benzyl isothiocyanate is a bioactive compound present in cruciferous vegetables, with anti-carcinogenic effects. It may target several molecular pathways. The compound is found to downregulate the NF-kB pathway, c-Met phosphorylation, p-Akt, uPA, p-62, mTOR, B lymphoma Mo-MLV insertion region 1 homolog (BMI1), aldehyde dehydrogenase 1 (ALDH1), KI-67, and survivin and upregulate p-53, p-73, liver kinase B1 (LKB1), Kruppel-like factor 4 (KLF4), Notch-2, Notch-4, and class O of fork head box transcription factors 1 (*FOXO1*), leading to inhibition of cell proliferation, metastasis, migration, invasion, and cell viability and induce apoptosis and autophagy [38]. The compound may also suppress the expression of cyclin B1 and CDK1 in BC cells, resulting in apoptosis and inhibition of tumor cell growth [123].

- Sulforaphane

Sulforaphane is an isothiocyanate present in cruciferous vegetables. It shows antioxidant and anti-inflammatory properties and targets several molecular pathways involved in the development of cancer. Sulforaphane possesses anti-proliferative, anti-metastatic, apoptotic, and chemo-sensitizing effects [38]. It inhibits the NF-kB pathway [124]. It downregulates DNMT1, DNMT3B, Akt, Bcl-2, Tumor necrosis factor-α (TNF-α), AMPK, MMP-2, MMP-9, MMP-13, COX-2, and p52 and upregulates Bax, p21, and p27 [38]. It is a potent epigenetic modulator and causes global DNA hypomethylation, histone acetylation, and alteration in micro-RNA profile, leading to its anti-cancer properties. It suppresses the expression of miR-92b, miR-23b, miR-381, and miR-382, resulting in cell cycle arrest and senescence [125]. Additionally, sulforaphane may increase the levels of carcinogen detoxifying enzymes such as NAD(P)H quinone dehydrogenase 1 (NQO1) and hem oxygenase-I (HO-I), decrease carcinogen activating enzymes including cytochrome P450 (CYP1A1, CYP1A2), enhance HDAC6, inhibit cancer stem cells, increase caspase activity, and may increase autophagy by promoting microtubule-associated protein light chain 3 (LC3-I and LC3-II) [126].

### 5.5. Others

- 3,3′-Diindolylmethane

3,3′-Diindolylmethane is a bioactive metabolite with phytoestrogenic properties. It can be obtained from indole-3-carbinol. It is found to suppress proliferation, migration, and invasion of BC cells by altering the expressions of several genes, including downregulation of epithelial–mesenchymal transition (*EMT*), N-cadherin, Akt, snail, slug, cathepsin B, cathepsin D, MMP-2, and MMP-9 and upregulation of p-21 and E-cadherin. The compound also decreased the expression of C-X-C motif chemokine receptor 4 (CXCR4), which is a receptor of CXCL12 chemokine associated with metastasis of BC cells via an ER-dependent pathway [127]. The compound may also promote G2/M cell cycle arrest and ROS production, leading to induction of apoptosis [128]. It may also inhibit the NF-kB pathway, leading to suppressed cell viability, colonization, and induction of apoptosis. The expression of FoxM1 may also decrease along with increase in miR-200 levels [129].

- α-Mangostin

α-Mangostin is a xanthone with antioxidant, anti-neoplastic, and anti-cancer properties. It can be derived from the pericarp of mangosteen. It is found to induce cell cycle arrest and apoptosis in various types of cancer [130]. The compound may downregulate myeloid leukemia cell differentiation protein (*Mcl*-*1)*, ER- α, HER2, ERK1/2, Bcl-2, PI3K, Akt, MAPK, FASN, and CDKs and upregulate p-p38, p-JNK1/2, p-53, Bax, PARP cleavage, caspase-3, caspase-7, caspase-8, caspase-9, and checkpoint kinase 2 (CHEK2), resulting in inhibition of cell proliferation and colonization and promote apoptosis. It is clinically important and may serve as a potential bioactive phytochemical for the treatment of BC. The main phytochemicals known for their anti-cancer effects are summarized in Table 1.

### 5.6. Clinical Trials

Numerous clinical studies have established the anti-cancer properties (which are currently used clinically) of various phytochemical compounds [131], the effects of curcumin involvement in anti-cancer therapy being extensively addressed in the published data. The systemic bioavailability of this compound, which apparently manifests itself at a low plasma concentration, is an additional problem in experimental research on the effects of curcumin in humans. Optimizing the bioavailability of curcumin using various drug delivery strategies provides more relevant results, highlighting that in vitro/in vivo anti-tumor activity can be repelled in the clinical context by addressing different pharmacological strategies [132,133]. 

In fourteen BC patients, in a phase I dose-finding trial, the curcumin was tested in combination with docetaxel, dose 100 mg/m^2^. Five (63%) of the eight patients who were evaluated for response had a partial response. A maximum tolerated dose of 6000 mg/day was established for one week, followed by two weeks without administration [134].

The efficacy and safety of intravenous curcumin, respectively, was evaluated in a phase II clinical trial in subjects with advanced, metastatic BC using the combination with paclitaxel. After 3 months of therapy, the combined curcumin–paclitaxel treatment was found to have superior effects to the paclitaxel–placebo combination in terms of overall response rate and physical performance. By intravenous administration of curcumin, there were no major safety problems, the quality of life was in good parameters, and a benefit was observed in reducing fatigue [135]. 

Additionally, in BC patients, a trial that used oral curcumin administration in a dose of 6 g daily proved a relevant decrease in radiotherapy-induced dermatitis, demonstrating an effective real prevention of dermatitis after radiation [136].

Although multiple clinical trials using some flavonoid compounds (i.e., flavopyridol, genistein, soy isoflavones, etc.) have led to the determination of appropriate doses/dose ranges without side or adverse effects in patients, there are still no reliable results supporting the inhibitory effect of a diet rich in substances of this type on BC [137,138]. Some of the most relevant clinical studies have investigated the use of the curcumin, genistein, and sulforaphane’s anti-tumor activity, respectively; their efficacy as therapeutic agents in different tumors are summarized in Table 2.

However, the published results of the recent clinical trials mentioned above have been shown to be insufficient to address these substances as standard anti-cancer therapy. Extensive, randomized studies are certainly needed to investigate the true effect of these phytocompounds in oncology.

## 6. Challenges in Clinical Applications 

The development of drugs from phytochemicals is a challenging task. Despite many successful preclinical studies, the number of clinical studies is inadequate to date, and results are often inconclusive. One of the major factors interfering with the clinical translation of the phytochemicals is their bioavailability [144]. Their quick metabolism, low solubility and permeability, and metabolic transformation are important limiting factors. Additionally, the poor biodistribution of the phytochemicals further challenges their clinical applications [145,146,147]. The exact molecular pathways and interactions with several signaling molecules involved in the biological activities of these phytochemicals are not completely understood yet [38]. Moreover, certain phytochemical supplements may also exhibit negative side effects and toxicity if they are not accurately standardized and specific doses are not established [24,53,148]. 

## 7. Authors’ Opinion

BC research is the major conceptual research being undertaken as the incident rate is increasing. It is not only seen in females, but also males have shown with this incidence and the progression of this disease. The major drawback when undergoing this research is its targeting and toxicity related issues. Patients experience post-treatment side effects such as hair loss, weight loss, and loss of appetite as after effects of the conventional therapy; additionally, the success rate of the therapy is less, and the recurrence rate is more. 

There are several issues raised by the use of natural ingredients, as follows: the high cost of some of the ingredients—synthetic ingredients have been produced, but they lack the potent/potentiating property of the original ones; abundance/dose administered—when compared to the conventional therapy, while using the natural medicine, the dose of the natural administered product is higher; and the change in the geographical cultivation area (altitude, climatic factors, soil proprieties, etc.) of the plants from which the respective product is extracted modifies the composition of the active ingredients, implicitly modifying/reducing their potentiating effect. 

When focused on the research point of view, the combination strategy can be adopted, with which the combination of the two natural sources can be used or the combination of one natural ingredient and a synthetic one. For this, the compatibility issue can play a major role in its progress. However, the combination approach can produce synergistic activity whereby it focuses on multiple pathways of the disease, effecting the treatment of BC. The present article aims to summarize the specific molecular pathways involved in the genetic effects of phytochemicals in BC. Phytochemicals are natural ingredients whose ability to treat various diseases has been proven. It has been reported that, with the potent activity of phytochemicals, the incident rate and the recurrence of BC could be minimized to an extent. On the other hand, phytochemicals are reported for the treatment and management of BC, but more clinical supportive data are needed for confirmation. The research on phytochemicals is a diverse field that needs to be studied; this review article gives researchers a background support for new discoveries and evolutions in the field of BC research.

## 8. Conclusions and Future Perspectives 

From the present review, it can be observed that phytochemicals do play important role in the prevention and management of BC. They are found to target various molecular pathways and alter the expression of several genes involved in tumorigenesis at transcriptional, translational, and post-translational levels. Phytochemicals upregulate the expression of tumor suppressors and downregulate the oncogenes. They prevent cell proliferation, differentiation, metastasis, migration, invasion, and angiogenesis, induce apoptosis, and sensitize cancer cells to chemotherapy and radiotherapy. They also cause epigenetic modifications of gene expression by altering DNA methylation, histone acetylation, and non-coding RNA levels. Such effects make them ideal leads for anti-BC drug development. 

However, the bioavailability and metabolism of these phytochemicals are not well established. Newer technologies such as nano-encapsulation, nano-emulsion, and other nano-formulation based drug delivery systems may be utilized for targeted delivery of such phytochemicals and increased efficiency. Huge amounts of unique phytochemicals are present in nature and, thus, in silico studies may further accelerate the identification process for drug development. These phytochemicals are also found to enhance the efficacy of conventional therapies. It is thus essential to evaluate the effectiveness of these phytochemicals in combination with various chemotherapeutics. Moreover, the exact molecular pathways and gene expression patterns associated with the beneficial effects of these phytochemicals should be determined by proper preclinical and clinical studies. 

## Figures and Tables

**Table 1:** Phytochemicals showing effects against breast cancer cell lines.

IUPAC Name	Structure	Phytochemical Usual Name/Natural Sources
Polyphenols		
[(2R,3R)-5,7-dihydroxy-2-(3,4,5-trihydroxyphenyl)-3,4-dihydro-2H-chromen-3-yl] 3,4,5-trihydroxybenzoate	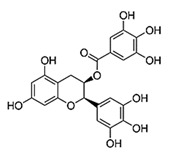	Epigallocatechin gallate/ Green Tea
5,7-dihydroxy-3-(4-hydroxyphenyl) chromen-4-one	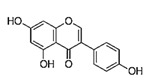	Genistein/Soybean, Soy based products
2-(3,4-dihydroxyphenyl)-3,5,7-trihydroxychromen-4-one	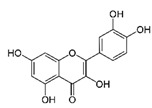	Quercetin/Apple, Grapes, Onion, Berries
5,7-dihydroxy-2-(4-hydroxyphenyl) chromen-4-one	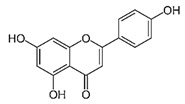	Apigenin/Grapefruit, Chamomile, Parsley, Celery
2-(3,4-dihydroxyphenyl)-5,7-dihydroxychromen-4-one	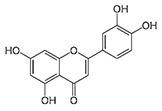	Luteolin/Parsley, Celery, Thyme
3,5,7-trihydroxy-2-(4-hydroxyphenyl) chromen-4-one	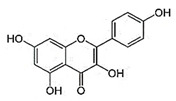	Kaempferol/Fruits and Vegetables
(E)-1-(2,4-dihydroxyphenyl)-3-(4-hydroxyphenyl) prop-2-en-1-one	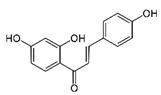	Isoliquiritigenin/Licorice, Soybeans
(1E,6E)-1,7-bis(4-hydroxy-3-methoxyphenyl) hepta-1,6-diene-3,5-dione	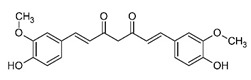	Curcumin/Turmeric
(2R,3R)-2,3-bis[(4-hydroxy-3-methoxyphenyl) methyl] butane-1,4-diol	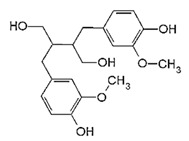	Secoisolariciresinol / Flax Seeds, Sesame Seeds, Sunflower Seeds
5-[(E)-2-(4-hydroxyphenyl) ethynyl] benzene-1,3-diol	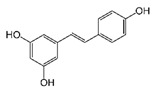	Resveratrol/Grapes, Wine, Blueberries, Cranberries, Mulberries
4-[(E)-2-(3,5-dimethoxyphenyl) ethynyl] phenol	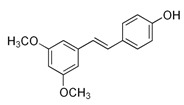	Pterostilbene/Blueberries
(2R,3R)-3,5,7-trihydroxy-2-[(2R,3R)-3-(4-hydroxy-3-methoxyphenyl)-2-(hydroxymethyl)-2,3-dihydro-1,4-benzodioxin-6-yl]-2,3-dihydrochromen-4-one	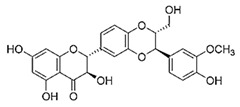	Silibinin/Milk Thistle Seeds
Terpinoids		
2-methyl-5-propan-2-ylcyclohexa-2,5-diene-1,4-dione	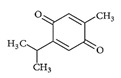	Thymoquinone/Black Cumin
(1S,2R,4R,7E,11S)-4,8-dimethyl-12-methylidene-3,14-dioxatricyclo [9.3.0.02,4] tetradec-7-en-13-one	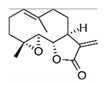	Parthenolide/Feverfew
Saponins		
(3S,5R,8R,9R,10R,14R,17S)-17-(2-hydroxy-6-methylhept-5-en-2-yl)-4,4,8,10,14-pentamethyl-2,3,5,6,7,9,11,12,13,15,16,17-dodecahydro-1H-cyclopenta[a]phenanthren-3-ol	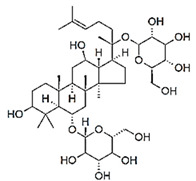	Ginsenosides/Ginseng
Isotiociantes		
1-isothiocyanato-4-methylsulfinylbutane	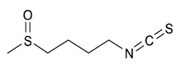	Sulforaphane/Broccoli
Iso-thiocyanato-methyl benzene	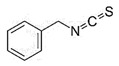	Benzyl isothiocyanate/Cruciferous Vegetables
Others		
3-(1H-indol-3-ylmethyl)-1H-indole	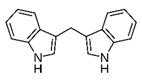	3,3′-Diindolylmethane/Cabbage
1,3,6-trihydroxy-7-methoxy-2,8-bis(3-methylbut-2-en-1-yl)-9H-xanthen-9-one	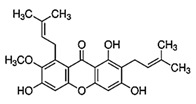	α-Mangostin/Mangosteen

**Table 2:** Selected clinical trials evaluating the effect of some phytochemicals in breast cancer (BC) patients.

Phytochemicals	Type of the Study/ No. Participants	Details of Breast Cancer	Outcomes	Ref.
Curcumin	Phase I/40	Advanced and metastatic	Dose range study	[134]
	Phase II/29	BC	Prevention	[139]
	Phase II/35	High risk	Dose range study	[140]
	Phase I/686	BC	Did not significantly reduce radiation dermatitis	[138]
	Phase II/30	BC	Reducing fatigue in patients with chemotherapy undergoing radiotherapy	[140]
	Phase II/150	Metastatic	Superior to the paclitaxel–placebo combination	[135]
Genistein (with Gemcitabine)	Phase II/17	Stage IV	No effect	[141]
Genistein	Phase II/126	BC	No effect	[142]
	Phase I	BC	Dose range study	[138]
Sulphoraphane/isoti	Phase II/60	Metastatic	Anti-tumor activity and prolonged disease stabilization	[143]
